# T Follicular Helper Cells in Autoimmune Disorders

**DOI:** 10.3389/fimmu.2018.01637

**Published:** 2018-07-17

**Authors:** Noémie Gensous, Manon Charrier, Dorothée Duluc, Cécile Contin-Bordes, Marie-Elise Truchetet, Estibaliz Lazaro, Pierre Duffau, Patrick Blanco, Christophe Richez

**Affiliations:** ImmunoConcept, UMR-CNRS 5164, Université de Bordeaux, Bordeaux, France

**Keywords:** auto-immunity, auto-immune diseases, T cells, T helper follicular cells, systemic lupus erythematosus

## Abstract

T follicular helper (Tfh) cells are a distinct subset of CD4^+^ T lymphocytes, specialized in B cell help and in regulation of antibody responses. They are required for the generation of germinal center reactions, where selection of high affinity antibody producing B cells and development of memory B cells occur. Owing to the fundamental role of Tfh cells in adaptive immunity, the stringent control of their production and function is critically important, both for the induction of an optimal humoral response against thymus-dependent antigens but also for the prevention of self-reactivity. Indeed, deregulation of Tfh activities can contribute to a pathogenic autoantibody production and can play an important role in the promotion of autoimmune diseases. In the present review, we briefly introduce the molecular factors involved in Tfh cell formation in the context of a normal immune response, as well as markers associated with their identification (transcription factor, surface marker expression, and cytokine production). We then consider in detail the role of Tfh cells in the pathogenesis of a broad range of autoimmune diseases, with a special focus on systemic lupus erythematosus and rheumatoid arthritis, as well as on the other autoimmune/inflammatory disorders. We summarize the observed alterations in Tfh numbers, activation state, and circulating subset distribution during autoimmune and some other inflammatory disorders. In addition, central role of interleukin-21, major cytokine produced by Tfh cells, is discussed, as well as the involvement of follicular regulatory T cells, which share characteristics with both Tfh and regulatory T cells.

## Introduction

T follicular helper (Tfh) cells represent a distinct CD4^+^ helper T cell subset, specialized for provision of help to B cells (1–4). They develop within secondary lymphoid organs (SLO) and can be identified based on their unique surface phenotype, cytokine secretion profile, and signature transcription factor. They support B cells to produce high-affinity antibodies toward antigens (Ag), in order to develop a robust humoral immune response and they are crucial for the generation of B cell memory. Whereas they are essential for infectious disease control and optimal antibody responses after vaccination, an excessive response can lead to a breakdown of tolerance. In this review, after introducing the biology of Tfh cells, we will consider in detail their role in the pathogenesis of autoimmune diseases and inflammatory disorders.

## Tfh Cells: Overview on Characterization, Generation, and Functions

T follicular helper cells were initially described in 2000 and 2001 in humans (1–4). They have been identified as a distinct T helper cell subset, based on their unique combination of surface markers [abundant expression of chemokine (C-X-C motif) receptor 5 (CXCR5) (1, 4), downregulation of C-C chemokine receptor type 7 (CCR7), and expression of the co-stimulatory molecules inducible co-stimulator (ICOS) (1) and programmed cell death protein-1 (PD-1)], cytokine production [expression of high levels of interleukin 21 (IL-21)], and specific transcription factor [expression of the nuclear transcriptional repressor B cell lymphoma 6 (Bcl-6)]. All are necessary for the development, the maintenance, and the functions of these cells. Tfh cells are crucial for the generation of germinal centers (GC) (5, 6), unique structures formed in SLO where antigen-primed B cells undergo proliferation, immunoglobulin (Ig) class switching, somatic hypermutation, and differentiation (7). Strong expression of CXCR5 by Tfh cells is pivotal for their migration into B cell follicles, in which they are attracted in response to the gradient expression of chemokine ligand 13 (CXCL13) (8–10). Within GC, Tfh cells are important regulators of B cell maturation and help signals rely on both cell-to-cell interactions and on the production of cytokines. Inside GC, Tfh cells are equipped with a specific combination of surface molecules, such as ICOS, a co-stimulatory molecule that belongs to the CD28 superfamily, PD-1, a potent inhibitory receptor, or CD40 ligand (CD40L), a member of the tumor necrosis factor family. Engagement of these molecules on their receptors on B cells delivers signals of survival, differentiation, or isotype switching (11–22). Tfh cells are also characterized by the secretion of a diversity of cytokines (23–26). Production of high levels of IL-21 is one hallmark of Tfh cells (23). This cytokine promotes B cells differentiation into plasma cells, somatic hypermutation, and Ig isotype switching (23, 27–30). Moreover, IL-21 promotes Tfh cells differentiation in a positive autocrine feedback loop (31–35).

Differentiation and development of Tfh cells is an extremely complex process, occurring in a multi-stage way. The essential transcription factor of Tfh cells, Bcl-6, controls their differentiation, their full maturation, and the expression of Tfh cells signature molecules (36–38). Bcl-6 expression, associated with the downregulation of its antagonist Blimp-1, leads to the inhibition of the other transcription factors specific to other T helper cell lineages (T-bet, GATA3, and RORγt specially). In addition to Bcl-6, other transcription factors, such as c-MAF, achaete–scute complex homolog 2, basic leucine zipper transcription factor (39), or IFN regulatory factor 4 are also crucial for Tfh cells differentiation (39–41). While Bcl-6 expression is essential for Tfh cells, it should be noted that it is not perfectly Tfh cell-specific, as it can be transiently up-regulated in dividing CD4^+^ T cells after activation by dendritic cells (42, 43). Micro-environmental factors are necessary for T cells to acquire a Tfh profile. First, Ag stimulation and constant initial interaction with Ag-presenting cells (APCs) and activated B cells play a crucial role in their differentiation from naive CD4^+^ T cells (2, 44–46). Second, a specific cytokinic milieu is necessary as IL-12, and to a lesser extent TGFβ, seem to be of particular importance for Tfh cells differentiation in humans (47–50). In addition, although originally recognized as an essential T-cell growth factor, IL-2 is a potent inhibitor of Tfh cells differentiation as the ligation of its receptor leads to STAT5 activation, promoting the formation of non-Tfh effector cells (51–53).

One layer of complexity in the study of Tfh cell populations is their substantial heterogeneity. Tfh cells are not a monolithic population with fixed phenotype and functions. They undergo changes over time in the expression of surface molecules and in the secretion of cytokines, in order to shape more efficiently the help delivered to B cells (45, 54, 55).

The biology of Tfh cells has been extensively studied in SLO in mouse models, but due to the difficulties of sampling in humans, different research groups have focused on identifying the circulating counterparts of bona fide Tfh cells in peripheral blood. Description of a CXCR5^+^ subset of memory CD4^+^ T cells has emerged few years ago (56–58) and has been subsequently referred to as circulating Tfh (cTfh) cells (59). Although these cells do not express Bcl6, they share phenotypic and functional properties with tissue Tfh cells (15, 56, 58–60). However, several questions are still under investigation regarding this population: their exact biology is still poorly defined, and how they relate to bona fide lymphoid Tfh cells remains unclear. Literature data, arising specially from SAP-deficient mice, suggest that development of cTfh cells does not require GC formation. These cells are committed to the Tfh lineage and are primarily generated before participating in GC reaction (54, 58). There is actually no consensus regarding phenotypic markers that should be used for the identification of this population. It is clear that other activated CD4^+^ T cell subsets, with other effector fates, can express transiently CXCR5, ICOS, or PD-1, but we can consider that sustained and/or high levels of expression of these molecules are characteristic of Tfh cells (59). The heterogeneous population of cTfh cells contains three major subsets, defined by their expression of the chemokine receptors CXCR3 and CCR6. They present similar properties with Th1, Th2, and Th17 cells: CXCR3^+^CCR6^−^ represent cTfh1 cells, CXCR3^−^CCR6^−^ cTfh2 cells and CXCR3^−^CCR6^+^ cTfh17 cells. cTfh2 and cTfh17 are more efficient than cTfh1 cells in helping B cells to proliferate and differentiate into plasma cells (56). Finally, within each of these subsets (cTfh1, cTfh2, or cTfh17), it is possible to subdivide other distinct subpopulations, according to the differential expression of the markers ICOS and PD-1 which can be used as indicators of active state of Tfh cells (58, 61, 62).

Regulatory pathways need to be engaged to control the development and maturation of Tfh cells, as well as their interactions with B cells. Within the diverse regulating mechanisms that we will not develop here, it is of particular importance to mention the role of another specific subset of CD4^+^Foxp3^+^ regulatory T cells, called follicular regulatory T (Tfr) cells, that has been more recently described. These cells share phenotypic characteristics with Tfh cells, with the expression of Bcl-6, CXCR5, PD-1, and ICOS. They are found within the GC, where they act to limit the size of the response, regulating the frequencies of both Tfh and GC B cells (63–66). Lack of Tfr cells is associated with greater GC reactions *in vivo*.

## Tfh Cells and Auto-Immunity: Generalities

Autoimmune diseases are caused by a breakdown of immune tolerance. Proliferation of self-reactive B cells with generation of high-affinity autoantibodies participate to the pathophysiology of these disorders and, in this way, have led to consider Tfh cells as possible actors in their pathogenesis. Moreover, in autoimmune diseases, inflammatory sites develop frequently lymphoid cell aggregations which include B and T helper cells, leading to the formation of ectopic lymphoid structures (67). These tissue-localized T–B-cell interactions which can promote the maturation of B cells and can potentiate the production of pathogenic autoantibodies (68), even if the exact nature of helper T cells in inflamed tissues is not completely understood currently.

Studies of the contribution of Tfh cells in autoimmune disorders have been initially limited to animal models, essentially because of difficulties in investigating Tfh cells from human SLO. The ulterior identification of circulating Tfh-like cells, which are more accessible than Tfh cells in tissues, has provided an opportunity to analyze these cells in the context of human autoimmune disorders. Since then, alterations in Tfh cells have been described in a broad range of autoimmune diseases, including systemic diseases, or organ- and cell-specific disorders. Increased numbers of cTfh cells, often correlating with the frequency of circulating plasmablasts or plasma cells, have been observed in several auto-immune diseases and alterations have also been reported regarding their polarization, their function, and the quality of help they provide. All the data collected so far in auto-immunity and in inflammatory diseases should not mask our lack of knowledge regarding the exact biology of cTfh cells and that analysis could be confounded by our incomplete understanding. Moreover, it is to mention that combination of markers used to define cTfh cell populations in the studies that will be mentioned in the next sections often differ among the laboratories. Some studies have defined cTfh cells as total CXCR5^+^CD4^+^ T cells, while other have investigated a subset of CXCR5^+^CD4^+^ T cells (CXCR5^+^ICOS^+^, CXCR5^+^PD1^+^, CXCR5^+^ICOS^+^PD1^+^, or CXCR5^+^IL-21^+^).

## Systemic Lupus Erythematosus (SLE)

Systemic lupus erythematosus is characterized by the presence of high-affinity pathogenic autoantibodies directed against nuclear components (69). Autoreactive B cells in SLE are derived from GC reactions (70); therefore, investigations of Tfh cell roles have been intensive since many years, both in murine models and in human SLE. In different mouse models sharing immunopathologic features with human SLE, it has been demonstrated that Tfh cells contribute to the activation of the immune system and to the pathogenesis of the disease. Animals in these different models have an aberrant expansion of Tfh cells, associated with dysregulation of the GC responses. BXD2 mice, which develop spontaneously autoantibodies and glomerulonephritis, are characterized by an increased frequency of PD1^+^ Tfh cells as compared to wild-type animals, correlating positively with the frequency of GC B cells and with levels of anti-dsDNA antibodies (71). Sanroque mice have a particular single recessive mutation in *roquin*, a gene encoding for a RING-type ubiquitin ligase family member that disrupts a repressor of ICOS expression. Animals are characterized by a SLE-like phenotype with lymphadenopathy and splenomegaly, high levels of anti-nuclear antibodies (AAN), autoimmune cytopenia, and immune complex deposits (72). Even without antigenic stimulation, a spontaneous formation of GC is observed, with an increase in the number of Tfh cells and an increase in their activity, particularly marked regarding the excessive production of IL-21 (72–75). The other murine model that has provided evidence of the implication of Tfh cells in the development of systemic autoimmunity is represented by BXSB mice, in which the duplication of the *Tlr7* gene promotes excessive signaling by self-autoantigens, leading to a severe inflammatory disease, with high levels of autoantibodies and proliferative glomerulonephritis (76). These animals have expanded numbers of B cells and Tfh cells, especially in the spleen (77). Besides, IL-21 is elevated in the serum and over-expressed in splenocytes (78). In this mouse model, IL-21R deficiency induces a decrease in the serum levels of AAN and prevents the apparition of renal disease (77). Moreover, therapeutic blockade of IL-21, by the administration of an IL-21R-Fc fusion protein, seems to have a biphasic response characterized by an aggravation of the disease when the treatment is given during early life and an anti-inflammatory effect (decrease in IgG1 serum levels, in proteinuria levels, and in histological glomerulonephritis) when it is administrated later in the disease course (79, 80). Implication of IL-21 in the pathogenesis of SLE is also supported by data obtained in two other murine models of the disease. In MRL-Fas(lpr) mice, accumulation of activated B cells, activated T cells, plasma cells, and spontaneous GC formation is dependent on IL-21R signaling. Administration of IL-21R-Fc fusion protein reduces disease severity (81, 82). Finally, in the NZB/NZW mouse model, blockade of IL-21R inhibits the progression of the pre-established disease (83). Murine models also point out the role of interactions between ICOS and its ligand in the development of systemic autoimmunity and suggest that this pathway could possibly be an interesting novel therapeutic target. In the NZB/NZW mice, blockade of ICOS pathway, by the use of a monoclonal antibody directed against ICOS-L, leads to the inhibition of the development of Tfh cells, to a decrease in anti-dsDNA antibody titers and to an improvement in kidney function (84, 85). Reduction in titers of anti-dsDNA antibodies is also observed in the mouse model MRL-Fas(lpr) when animals have an additional genetic deletion in ICOS (86). Finally, in B6.Sle1 mice, elevated expression of ICOS contributes to the expansion of Tfh cells and to the breakdown in peripheral tolerance (87).

Several lines of evidence also support a pathogenic role of Tfh cells and IL-21 in human SLE (Table 1). Data are available mostly for cTfh cells, which have been showed to be increased and have an active phenotype in SLE patients as compared to controls (88–98). This activated phenotype correlates with the number of circulating plasmablasts and with the levels of pathogenic autoantibodies (58, 88, 90, 91, 93–95, 98), but the correlation with disease activity observed in some studies (58, 90, 92, 97) has been considered inconsistent by other research teams (88, 94). Of particular interest are the observations made on alterations in the composition of cTfh cells subsets in SLE, associated with disease activity (99). Ratio of cTfh2 and cTfh17 (which are both considered as efficient B helpers) over cTfh1 are increased in SLE patients as compared to controls and disease activity correlates with the frequency of cTfh2 cells (99). Higher plasma levels of IL-21, as well as an increase in the frequency of IL-21-expressing CD4^+^ T cells, are found in SLE patients as compared to controls, correlating with the number of switched memory B cells and with several markers of disease severity (89, 91, 95, 100–104). Moreover, Tfh cells have been also investigated at tissue level in SLE: Tfh-like cells are present in inflamed kidney of patients with nephritis (105, 106), where they cluster with B cells building structures similar to ectopic GCs (106).

**Table 1:** T follicular helper (Tfh) cells in human SLE.

Study	Patients (*n*)	
**Circulating Tfh**		
He et al. (58)	26	Expansion of cTfh cells
Simpson et al. (88)	46	Expansion of cTfh cells No association with disease activity. Correlation with the titers of autoantibodies
Terrier et al. (89)	25	Expansion of cTfh cells
Feng et al. (90)	42	Expansion of cTfh cells Correlation with disease activity and with the titers of autoantibodies
Wang et al. (91)	23	Expansion of cTfh cells
Yang et al. (92)	30	Expansion of cTfh cells in patients with active SLE Correlation with disease activity
Zhang et al. (93)	29	Expansion of cTfh cells Correlation with the titers of autoantibodies
Choi et al (94)	57	Expansion of cTfh cells Correlation with disease activity and with the titers of autoantibodies
Szabo et al. (95)	25	Expansion of cTfh cells Correlation with the titers of autoantibodies
Umeda et al. (96)	64	Expansion of cTfh cells
Xu et al. (97)	40	Expansion of cTfh cells Correlation with disease activity and with the titers of autoantibodies
Huang et al. (98)	70	Expansion of cTfh cells Correlation with disease activity and with the titers of autoantibodies
Le Coz et al. (99)	23	Expansion of cTfh2 cells in patients with active SLE. Decrease in Tfh1 cell subset percentage
**Tissue level**		
Liarski et al. (105)	42	Identification of functional Tfh cells in lesions of lupus nephritis. Close proximity between B cells and Tfh cells
Chang et al. (106)	68	Presence of T:B cell aggregates or GCs in the tubulointerstitial infiltrate of lupus nephritis

*cTfh cells, circulating Tfh cells; GC, germinal center; SLE, systemic lupus erythematosus*.

The role of Tfr in SLE remains unclear and literature data are inconsistent. Some authors observed a reduction in the frequency of circulating Tfr cells in SLE patients (107, 108) with an increase in the ratio Tfh/Tfr, associated with a negative correlation with disease activity and titers of anti-dsDNA antibodies (107). On the contrary, another study revealed a significant increase of Tfr cells in peripheral blood with an increase in the Tfr/Tfh ratio (109). Discrepancies could be related to the strategy used to define Tfr cells by flow cytometry (CD4^+^CD25^+^CD127^low-intermediate^CXCR5^+^ (107) or CD4^+^CXCR5^+^FoxP3^+^ (109)), as well as the different recruitment of subjects within each study.

T follicular helper cell expansion and aberrant GC responses play clearly a crucial role in the pathogenesis of lupus in mice and data obtained so far in human SLE seem to go in the same direction. Tfh cells could, therefore, represent an interesting therapeutic target. Conventional treatments used in SLE (steroids, immunosuppressive drugs) result in a decrease in the number of activated cTfh cells (90, 91), associated with a clinical improvement (90). A treatment with low-dose recombinant human IL-2 in SLE patients leads also to a decrease in the relative number of cTfh cells (110). However, the specific targeting of these cells requires a better understanding of the mechanisms involved in their aberrant activation during SLE. In this way, OX40 ligand (OX40-L)–OX40 axis has been identified as a key player in the increased Tfh responses (111): OX40-L, highly expressed by myeloid APCs in active SLE patients in response to TLR7 activation by RNP-anti-RNP immune complexes, promotes the differentiation of CD4^+^ T cells in functional Tfh cells (111). In this way, in human SLE, the OX40-L–OX40 axis provides an amplification loop in the generation of autoantibodies (112) and could represent an attractive pathway to target. However, although anti-OX40 and anti-OX40L antibodies exist, there is for instance no ongoing trial of these molecules in human SLE.

## Rheumatoid Arthritis (RA)

Rheumatoid arthritis is a systemic auto-immune disease, associated with acute and chronic synovial inflammation, cartilage lesions, and bone erosion (113). Various autoantibodies associated with RA and which play important roles in the pathological progression of the disease have been identified, including rheumatoid factor and antibodies against cyclic citrullinated peptides (anti-CCP). Exact pathogenic processes of RA are not totally understood and involve many types of immunocompetent cells. Nevertheless, interactions between T and B cells are required for antibody production and, similarly to what are observed during SLE, many of the RA-related autoantibodies are high-affinity IgG antibodies, indicative of the involvement of GC reactions and, therefore, suggesting a critical role of Tfh cells in the disease pathogenesis.

Data from murine models of arthritis seem to indicate that some essential Tfh cells associated molecules are required for the disease development. For example, in the model of collagen-induced arthritis (CIA), CXCR5 was identified as an essential factor for the induction of the inflammatory autoimmune arthritis, as CXCR5-deficient mice and mice selectively lacking CXCR5 on T cells are resistant to CIA (114). In another model (transfer of self-reactive CD4^+^ cells from KRN-TCR transgenic mice into recipient animals), Chevalier et al. observed that deficiency in CD4^+^ cells of signaling of lymphocytic activation molecule-associated protein (SAP) (a protein known to promote Tfh cell differentiation during GC formation) protects mice from the induction of arthritis, indicating that long-lasting interactions between T and B cells and GC formation are required for the development of the disease (115).

In human RA, it is worth noting that literature data are conflicting regarding Tfh cells frequencies (Table 2). In five studies, it was observed increased frequencies of cTfh cells in patients as compared to controls (116–120), especially in those with new-onset disease. For some authors, this increase is constitutive and is not associated with the activity of arthritis, as both patients with active or inactive disease have higher frequencies of activated cTfh cells and of subsets with B cell helper phenotype (Tfh2 and Tfh17) (116). Still these findings contrast with other reports where percentages of cTfh cells have been associated with the disease activity [mostly assessed by 28-joint count disease activity score (DAS28)] (117–119, 121) and with the levels of autoantibodies (119–121). An enrichment of Tfh cells was observed in synovium tissues of patients with RA whereas they were absent in both osteoarthritis and normal synovium tissues (122, 123). By contrast of the five studies mentioned above, three other ones dismiss the presence of increased frequencies of Tfh cells during RA. No differences were found in the percentages of cTfh cells or within Tfh1, Tfh2, and Tfh17 subsets in RA patients, as compared to healthy controls or patients with undifferentiated arthritis, without correlation with DAS28 (124, 125) and Penatti et al. even observed lower frequencies of ICOS^+^ cTfh cells in patients with RA (122).

**Table 2:** T follicular helper (Tfh) cells in human RA.

Study	Patients (*n*)	
**Circulating Tfh**		
Arroyo-Villa et al. (116)	34	Expansion of cTfh cells
Zhang et al. (117)	54	Expansion of cTfh cells Correlation with disease activity (values of DAS 28)
Nakayamada et al. (118)	108	Expansion of cTfh cells in patients with active RA Correlation with autoantibody titers Abatacept treatment resulted in decrease in the proportion of activated cTfh cells
Wang et al. (119)	25	Expansion of cTfh cells Correlation with disease activity (values of DAS 28)
Ma et al. (120)	53	Expansion of cTfh cells Correlation with autoantibody titers (level of anti-CCP Ab)
Liu et al. (121)	120	Expansion of cTfh cells Correlation with disease activity (values of DAS 28) and with the titers of autoantibodies (anti-CCP Ab levels)
Penatti et al. (122)	25	Lower frequencies of cTfh cells ICOS+ in patients with RA
Costantino et al. (124)	24	No differences in cTfh cells between patients with RA and healthy controls or patients with undifferentiated arthritis
Chakera et al. (125)	35	No differences in cTfh cells between patients with RA and healthy controls
**Synovium tissues**		
Penatti et al. (122)	25	In the synovial compartment of RA patients, enrichment in Tfh cells as compared to patients with osteoarthritis
Chu et al. (123)	2	Localization of ICOS+ Tfh cells in the RA synovium tissues, as compared to osteoarthritis synovium or normal synovium tissues where they are absent (triple-fluorescence immunostaining and confocal laser scanning)

*Ab, antibody; Anti-CCP, antibodies against cyclic citrullinated peptides; cTfh cells, circulating Tfh cells; CXCR5, chemokine (C-X-C motif) receptor 5; DAS, disease activity score; ICOS, inducible co-stimulator; RA, rheumatoid arthritis*.

Investigations on the implication of Tfh cells in the pathogenesis of the disease have also led to the evaluation of the impact of their signature cytokine, IL-21. RA patients have higher serum levels of IL-21 as compared to controls (117, 120, 121), correlating with DAS28 (117, 121), serum anti-CCP antibodies, and frequencies of cTfh cells (121). IL-21R is highly expressed in inflamed synovial tissues of RA patients (126, 127), by macrophages but also by fibroblasts with activated phenotype (126). IL-21 has an impact on local T-cell activation and proliferation, but also promotes the aggressive migration, invasion, and metalloproteinase secretion by fibroblast-like synoviocytes (128). In RA synovial cell cultures, neutralization of IL-21 and IL-15 inhibits their pro-inflammatory cytokine production (129). Finally, in the K/BxN mouse model of RA, IL-21R deficiency is sufficient to protect from arthritis (130).

Potential use of Tfh cells as biomarkers of RA has conducted to the evaluation of the impact of treatments on these populations. In 2013, Wang et al. observed a significant reduction in the percentages of ICOS^+^ Tfh cells following 1 month of disease-modifying antirheumatic drugs (DMARDs) and *T. wilfordii* (Chinese herb) in the drug-responding patients (119). On the contrary, no decrease in the frequency of Tfh cells in response to 24 weeks of DMARD therapy (mostly methotrexate monotherapy) was observed, whereas IL-21 concentrations were remarkably reduced (117). In patients with RA treated by abatacept, which modulates CD28-mediated T cells co-stimulation, the treatment is associated with a marked decrease in the proportion of activated cTfh (118). Data from murine model of RA associated with a breach of self-tolerance brought some mechanistic explanations on this observation made in RA patients: abatacept is associated with a failure of T cells to acquire a follicular helper cell phenotype (CXCR5^+^ICOS^+^), to proliferate, to enter B cell follicles, leading to a reduction in antibodies responses (131).

It is known that environmental factors are implicated in the development of arthritis and two recent studies suggest that generation of RA-related Tfh cells possibly relies on gut microbiota. Using the K/BxN autoimmune arthritis model, Teng et al. evaluated the role of a specific type of commensal gut bacteria, named segmented filamentous bacteria (SFB), in the regulation of Tfh cell responses (132). SFB are known to drive autoimmune arthritis in this mouse model (133) and it was shown that SFB induce an increase in the systemic Tfh cell populations by driving differentiation and egress of Tfh cells from intestinal Peyer’s patches into systemic lymphoid sites, leading to an increase in auto-antibody responses and to the development of arthritis (132). Using the same murine models, Block et al. confirmed the requirement of gut microbiota in the differentiation of Tfh cells, the formation of GCs, the auto-antibody production, and the development of the experimental arthritis. Depletion of gut microbiota in the animals, by the use of antibiotics, reduced the number of Tfh cells and the levels of antibodies (134).

Finally, we would like to highlight the results published recently by Rao et al. in Nature, who identified a novel subset of T cells in the synovium of patients with RA (135). Using multidimensional cytometry, transcriptomics, and functional assays to analyze activated T cells in joint tissues, authors observed, in seropositive RA patients, abundant populations of a specific subset of T cells, defined as PD-1^hi^CXCR5^−^CD4^+^ and which they named “peripheral helper” cell population. This population, not expanded in patients with seronegative RA and of whom frequencies are highly correlated with disease activity in response to therapy, shares B-cell-helper functions with Tfh cells but seems to differ from them by a specific migratory program targeting inflamed tissues rather than lymphoid tissues (135). These results need to be confirmed and some questions need to be addressed, but this study raises the possibility of the existence in RA of a subset with high pathologic abilities that could be of great interest in a therapeutic perspective.

T follicular helper cells have also been investigated in patients with ankylosing spondylitis (AS), another rheumatologic disease, with contradictory results. Higher frequencies of cTfh cells have been observed in AS patients as compared to controls, associated with elevated concentrations of serum IL-21 (136, 137), whereas Bautista-Caro et al. reported decreased frequencies of cTfh cells, plasmablasts and underrepresentation of cTfh subsets with a B helper phenotype in patients with AS naïve from TNF blockers (138). Frequencies of Tfr cells were also reported to be significantly higher in AS patients than in healthy controls, with higher ratio of Tfr/Tfh cells (137).

## Sjogren Syndrome

Primary Sjögren’s syndrome (pSS) is a systemic autoimmune disease, presented as a disabling sicca syndrome associated with asthenia and pain (139). It is characterized by lymphocytic infiltration and destruction of exocrine glands, primarily the lacrimal and salivary ones. Crucial role of B cells in pSS pathogenesis is illustrated by the emergence of circulating immune complexes, autoantibodies, ectopic GCs in the affected tissues, and enhanced risk of developing B cell lymphoma (140) underlying a possible important role played by Tfh cells in the pathogenesis of this disease. Increased percentages of cTfh cells (especially cTfh17 subset) have been observed in peripheral blood of patients with pSS (141, 142). This increase seems to be limited to pSS patients with severe manifestations of the disease. Indeed, in patients with pSS who present extraglandular manifestations (EGMs), Szabo et al. observed an increase in activated cTfh cells (ICOS^+^ or PD1^+^ cTfh) as compared to pSS patients without EGMs and to healthy controls, whereas there was no difference in cTfh percentages between patients without EGMs and controls (143). Elevated Tfh percentages were also observed in the anti-SSA/SSB positive patients (143). Similarly, a significant elevation in the proportion of IL-21-producing PD1^+^ cTfh cells was described in pSS patients with EGMs and in patients with autoantibodies against anti-SSA/Ro (95). More interestingly, correlation between cTfh cells percentages and importance of glandular involvement assessed by the focus scores of labial salivary gland biopsies has been estimated with a coefficient R equal to 0.6984 (143). Otherwise, increased serum levels of IL-21 are observed in pSS patients with a significant association with the systemic DAS28 (ESSDAI) (144, 145). Regarding salivary glands, Jin et al. reported increased numbers of Tfh cells in pSS patients as compared to controls (141). When analyzing the expression of cytokines and transcription factors associated with the different Th subsets (Tfh, but also Th1, Th2, and Th17) in labial salivary glands, Maehara et al. observed an increase in the expression of all Th subset-related molecules in pSS patients as compared to controls. However, expression of Tfh-related molecules was associated with a strong lymphocytic infiltration and especially in the presence of GCs (146). Differentiation of naïve CD4^+^ T cells seems to be particular during pSS and implies the interactions with salivary gland epithelial cells such as IL-6 and ICOS-L expressed by these cells contribute to the direct induction of Tfh cells differentiation (145).

Frequency of cTfh cells in pSS patients is almost reduced by 50% after B cell depletion therapy and reach levels comparable to controls, in association with a significant lowering of serum levels of IL-21, with a decrease in anti-SSA/Ro and antiSSB/La antibodies and with the improvement of the disease activity measured by ESSDAI (147). During B cell repopulation, frequencies of cTfh cells return to baseline levels. Like described in RA, specific effects of abatacept on Tfh cells are observed during pSS treatment (148). Abatacept results in the predominant reduction of percentages and numbers of cTfh cells (other effector subsets are not affected, except Treg cells), of serum levels of IL-21 and of autoantibodies. A decrease in ICOS expression on CD4^+^ T cells is observed in both peripheral blood and parotid gland tissue and this phenomenon correlates significantly with a lowering in the DAS28 (148).

## Neurological Diseases

### Multiple Sclerosis (MS)

Multiple sclerosis is a chronic, inflammatory, and autoimmune disease affecting the central nervous system (CNS), leading to the destruction of myelin and axons (149). In pathological studies, important meningeal inflammation with ectopic lymphoid follicles, B and plasma cells is observed (150, 151). This suggests a possible implication of T helper cells and especially Tfh cells in the pathogenesis of the disease. In peripheral blood from patients with relapsing–remitting or secondary progressive forms of the disease, an increase in the frequencies of ICOS^+^ cTfh cells is observed, associated with an increased expression of Tfh and plasmablast markers by cerebrospinal fluid (CSF) cells (152). Activated memory cTfh cells (CCR7^+^ICOS^+^) were also found increased in another study in patients with relapsing MS. Moreover, they observed increased levels of plasma and CSF IL-21 correlating positively with the score of severity of the disease (EDSS score) and with levels of auto-antibodies directed against myelin basic protein or myelin oligodendrocyte glycoprotein (153). Treatment with systemic steroids (methylprednisolone) leads to a decrease in the numbers of activated cTfh cells and in the plasmatic levels of IL-21 (153).

### Neuromyelitis Optica Spectrum Disorders (NMOSDs)

Neuromyelitis optica spectrum disorders are also inflammatory demyelinating diseases affecting CNS, characterized clinically by attacks of myelitis and optic neuritis and biologically by the presence of highly specific and pathogenic autoantibodies directed against the extracellular domain of the water channel protein aquaporin-4 expressed in astrocytes (NMO-IgG) (154). Activated Tfh cell frequencies are higher in NMOSD subjects, as compared to healthy controls and even as compared to MS patients and are associated with disease activity and with increased levels of plasma and CSF IL-21 (155, 156). Treatment with methylprednisolone is associated with a significant decrease in the proportion of cTfh cells in NMOSD patients (155).

### Myasthenia Gravis (MG)

Myasthenia gravis is characterized by the production of anti-acetylcholine receptor (AchR) antibodies, leading to a dysfunction of the neuromuscular junction and *in fine* to muscle weakness (157). Ocular and generalized MG are the two major clinical forms of the disease. Expansion of cTfh cells, concomitant with expansion of circulating plasmablasts, is observed in patients with MG as compared to healthy control subjects, and is associated with the clinical severity and the form of the disease (158–161). Strong correlations between cTfh cells and titers of anti-AChR autoantibodies are frequently described (159–161). On the contrary, proportions of Tfr cells are decreased in MG patients as compared to controls, leading to an imbalance in the Tfr/Tfh ratio (159, 162). One important specificity of MG is the frequent association with thymoma and the use of thymectomy as a treatment, leading to the possible pathological analysis of thymus obtained from MG patients. Clinical severity of the disease (subjects with generalized MG versus subjects with ocular MG and controls) is correlated with higher mRNA expression of four markers by thymoma cells (CXCR5, Bcl-6, ICOS, and IL-21) (163). Higher percentages of thymic Tfh cells are present in MG patients, as compared to patients with thymoma without MG or patients with healthy thymus (control thymic biopsies from cardiac surgery cases) (164).

## IgG4-Related Disease (IgG4-RD)

IgG4-related disease consists in lymphoplasmacytic infiltrates of CD4^+^ T cells and IgG4^+^ plasma cells associated with fibrosis. It affects mainly males over 50 years and may involve all tissues (pancreatitis, retroperitoneal fibrosis…) (165). IgG4-RD patients are characterized by higher proportions of cTfh cells, as well as increased proportions of plasmablasts as compared to controls (166). The two populations correlate positively within each other and cTfh cell rates are positively correlated with EGMs of IgG4-RD (166). Proportions of cTfh cells that express a high level of PD-1 positively correlate with serum levels of IgG4, IgG4/IgG ratio, and number of involved organs (167). When cTfh subsets are analyzed, a significant specific expansion of Tfh2 cells is observed, associated with serum levels of IgG4, IgG4/IgG ratio, number of plasmablasts, disease activity, and number of affected organs (168–170). Tfh2 cells appear to be responsible, in the pathogenesis of the disease, for the induction of the differentiation of naïve B cells into plasmablasts and of the increase in the production of IgG4 in patients with active disease (169). Contradictory results have been published regarding the population of Tfh1 cells: a Japanese team observed an increase in the number of activated Tfh1 cells in IgG4-RD, correlating with disease activity but not with serum IgG4 levels, whereas another team reported a decrease in the proportion of cTfh1 cells in patients as compared with controls (170).

One particularity of IgG4-RD is the existence of numerous data with histopathological examination. In fact, in IgG4-RD, it is necessary to get a biopsy-proven diagnosis, allowing the analysis of Tfh cells directly in the involved tissues. Histological data reveal marked Tfh cell infiltration or overexpression of Tfh-related molecules in the different affected organs studied (166, 171–173), colocalizing with B cells and plasma cells (171). Tfh cells infiltrating the submandibular glands (SMGs) of patients suffering from IgG4 dacryoadenitis and sialadenitis are characterized by high expression levels of Bcl6, PD-1, and ICOS, as compared with SMGs from control patients with head and neck cancers, and functional analysis reveal that they have a higher capacity than tonsillar Tfh cells to help B cells to produce IgG4 (167). Expression of IL-21 in labial salivary glands from patients with IgG4-RD correlates with the number of GCs and with the IgG4/IgG ratio (172). All teams working on Tfh cells and IgG4-RD showed a decrease in Tfh percentages in patients treated by steroids or rituximab, correlating to the improvement of symptoms (166, 167, 169, 170).

## Vasculitis

### Large Vessels Vasculitis

To our knowledge, role of Tfh cells in the physiopathology of large vessels vasculitis has been rarely investigated. One team studied Tfh cells in granulomatosis with polyangiitis (GPA) (174), disease characterized, in the majority of patients, by the development of ANCA autoantibodies with specificity for PR3 (ANCA/anti-PR3). Because GPA has benefited recently from B-cell depletion therapy, it raises the question of the underlying mechanism of its effectiveness. Authors found, in GPA patients on conventional therapies, an increased frequency of cTfh cells. This increase is not observed in GPA patients treated with rituximab who are clinically improved and in whom cTfh cells frequencies are statistically not different from those seen in healthy controls (174).

### Small Vessels Vasculitis

Ig A vasculitis, also called Henoch Schönlein Purpura (HSP), is the most common small vessel vasculitis which affects particularly children. Vascular deposits of IgA-related immune complexes characterize this systemic inflammatory disease. Circulating Tfh cells are increased in children with acute HSP as compared to healthy controls and are associated with higher levels of IL-21 (175, 176). Levels of cTfh and IL-21 lowered significantly following disease remission (175). Adult patients with HSP nephritis (HSPN) display same features, with increased levels of cTfh cells and IL-21 as compared to healthy controls and a significant reduction after treatment (177). When further investigating Tfh subpopulations, an increase in cTfh2 and cTfh17 is present in HSP patients compared to healthy controls and their levels positively correlated with serum IgA ones (178). The increases in cTfh2 and cTfh17 cells counts are abrogated by treatment.

## Idiopathic Thrombocytopenic Purpura (ITP)

Idiopathic thrombocytopenic purpura is characterized by a low platelet count, which is the result of both insufficient platelet production and increased platelet destruction by auto-antibodies directed against platelet glycoproteins. Main antibody isotype in ITP is represented by IgG, which underlies a class-switch recombination, a mechanism supported by Tfh cell help to B cells. In adult ITP patients, there is an increase in the proportion of cTfh cells with high expression of ICOS or PD-1 (179). This is particularly marked in the sub-group of patients with positivity of anti-platelet antibodies as compared to platelet-antibody-negative patients. Plasma levels of IL-21 are also significantly increased in patients with active ITP as compared to controls (179, 180). In the pediatric population, which is frequently impacted by ITP, similar results are observed: frequencies of cTfh cells are markedly increased during ITP, with a strong negative correlation between the proportion of cTfh cells and platelet count, as well as with increased serum levels of IL-21 (181). It should be highlighted that the frequencies of cTfh cells return to normal levels after therapy (intravenous Ig, corticosteroids, or both) in patients with newly diagnosed ITP, whereas children who fall in chronic ITP (cITP) have a persistent increase in both cTfh cell frequencies and IL-21 levels. Moreover, in cITP subjects, overexpression of ICOS-L by CD19^+^ B cells remains after treatment, while it markedly decreases in the other group (181).

Spleen is the site of platelet destruction by splenic macrophages phagocytosis and as it is the primary site for the activation of B cells and for the autoimmune response and, therefore, an organ of interest in ITP. Splenectomy is one possible therapeutic option for resistant ITP and pathological analysis of the resected organs gives precious information. Audia et al. characterized Tfh cells in the spleen of 13 ITP patients compared to 8 controls (splenectomy because of spleen traumatism) (182). Splenic Tfh cells frequencies (CXCR5^+^ICOS^+^PD-1^hi^) are increased during ITP, concomitant with an expansion of GCs and with an increase in splenic CD38^+^ B cell subsets (pre-GC B cells, GC B cells, and plasma cells). Furthermore, IL-21 expression in splenic CD4^+^ T cells correlates with Tfh cell abundance. Finally, *in vitro* stimulation of B cells with IL-21, in the presence of CD40 engagement, induces the differentiation of B cells in antiplatelet antibodies secreting plasmablasts in ITP patients (182). The expansion of splenic Tfh cells is dramatically decreased after B cell depletion induced by rituximab, associated with a decrease in the absolute count of cTfh cells, even if the therapy is not clinically effective (183).

## Chronic Inflammatory Skin Diseases

### Psoriasis

Skin lesions in psoriasis are thought to be due to a deregulated interplay between immune cells and keratinocytes, leading to proliferation of keratinocytes in the interfollicular epidermis, inflammation of the stratum corneum, dermal angiogenesis, and infiltration with mononuclear cells. Circulating Tfh cells are increased in blood from patients with psoriasis vulgaris as compared to controls, both from a numerical but also from an activation point of view, and frequencies of some activated counterparts of cTfh cells are correlated with disease severity [Psoriasis Area and Severity Index (PASI) score] and with the accumulation of activated B cells (184, 185). Regarding cTfh cells subpopulations, psoriatic patients are characterized by a significant increase in cTfh17 rates, with a trend of increasing frequency of cTfh2 cells and decreasing frequency of cTfh1 cells, leading to an increase in (Tfh17^+^Tfh2)/Tfh1 ratio (186). Frequency of cTfh17 correlates with the PASI score and decreases with the improvement of the skin disorder (186). Frequencies of cTfh cells are reduced after 1 month of acitretin treatment (184). In psoriatic skin lesions, Tfh are found in great amount contrary to healthy skin tissues or non-lesional skin tissues of psoriasis (184).

Serum levels of IL-21 are higher in patients with psoriasis than in controls and positively correlate with PASI score (185). Moreover, high levels of IL-21 protein and IL-21 mRNA are observed in lesional psoriasis skin compared to samples taken from non-lesional skin of the same patients and from healthy controls (187).

### Atopic Dermatitis (AD)

Because of an increased level of serum IgE in AD, some authors have focused on Tfh cells in this disease. There is an increased level of ICOS^+^Tfh and ICOS^+^PD1^+^ Tfh cells in children with AD when compared to adults with AD and to controls. Despite no difference in serum levels of IL-21, absolute numbers of IL-21-producing Tfh cells are significantly expanded in children with AD, and correlate to disease activity (measured by the SCORAD index) (188).

### Pemphigus and Bullous Pemphigoid

Pemphigus is an autoimmune disease with antibodies directed against the desmosomal cadherin Desmoglein (Dsg) 3 and Dsg1 that cause loss of keratinocyte adhesion in the human skin. There is an increase in percentage of total cTfh in peripheral blood of patients with pemphigus, along with elevated levels of serum IL-21, despite no difference in ICOS^+^ or PD1^+^ Tfh cells between patients and healthy controls (189). By stimulating PBMC from pemphigus patients and controls with Dsg3 protein *ex vivo*, authors identified autoreactive IL-21-secreting cells in 50% of the pemphigus patients.

Bullous pemphigoid is another autoimmune skin disease, with antibodies directed against hemodesmosomal proteins within the dermal–epidermal junction [non-collagenous 16A domain (NC16A) and transmembrane domain of the hemidesmosomal protein (BP180)]. Increased level of IL-21 and increased frequency of ICOS^+^ and PD1^+^ cTfh cells are positively correlated with high levels of serum anti-BP180-NC16A antibodies, which have been recognized as a serum marker of disease severity. Levels of serum IL-21 and Tfh rates are significantly reduced after efficient therapy with methylprednisolone (190).

## Endocrinopathies

### Type 1 Diabetes (T1D)

Type 1 diabetes is due to the destruction of insulin-producing-ß-cells in the pancreas. Despite being considered as a T-cell-driven disease, a pathogenic role of B cells producing autoantibodies against ß-islets has been proven. Using a transgenic mouse model of diabetes, Kenefeck et al. identified a Tfh signature (that is a strong up-regulation of Tfh cell genes) in the islet-specific T cells responding to pancreatic antigen, as compared to T cells sorted from inguinal lymph nodes (191). This was confirmed at the protein level by flow cytometry. Interestingly, transfer of T cells with a Tfh cell phenotype (obtained from pooled pancreatic lymph nodes from diabetic mice and enriched according to CXCR5 expression) transferred diabetes to recipient animals more efficiently than CXCR5-depleted T cells (191).

At the transcriptomic level, memory CD4+ T-cells from T1D patients (with a mean duration of T1D of 19 years) overexpress Tfh cell genes (*CXCR5, ICOS, PD-1, Bcl6*, and *IL-21*) (191). Rates of cTfh cells are increased in patients with T1D, as compared to controls (192–194). This increase is associated with an enhanced IL-21 production by memory CD4^+^ cells in patients (192, 193). Interestingly, authors have shown an increase in activated cTfh (CXCR5^+^PD1^+^ICOS^+^) cells in newly diagnosed T1D children and in at-risk children with impaired glucose tolerance compared to aged-matched healthy children (194). A longitudinal analysis of at-risk children showed an increased rate of Tfh just before progression to clinical T1D, whereas children who did not progress had a stable Tfh frequency. Comparison between diabetic patients with ≤1 autoantibody and diabetic patients with ≥2 antibodies showed a strongly increased frequency of activated cTfh cells in the second group. It suggests that multiple autoantibody-positivity identifies a subgroup of patients with increased Tfh activation at disease presentation (194).

PD1^+^ Tfh cells positively correlate with blood glucose levels and negatively to the Glomerular Filtration Rate (eGFR) in patients with diabetic nephropathy (195). Besides, frequency of ICOS^+^ Tfh cells seems to inversely correlate with the concentrations of fasting serum C-peptide, while after 4 months of rituximab therapy, 50% of patients increase their levels of this marker, parallel to the decrease in Tfh percentages (192).

### Autoimmune Thyroid Disease

Elevated percentages of ICOS^+^ and PD1^+^ Tfh cells are present in patients with autoimmune thyroid diseases [Grave disease (GD) or Hashimoto’s thyroiditis (HT)]. ICOS^+^ cTfh cells correlate positively with the serum concentrations of autoantibodies directed against anti-TSH receptor, thyroperoxidase, or thyroglobulin (196).

Analysis of thyroid tissues is sometimes possible in these diseases: both GD and HT are characterized by an increased lymphocytic infiltration (196–198), with frequent GCs formation. Tfh cells are detected *in situ*, in HT (196) and GD (197). Expression of Tfh-related molecules (IL-21/IL-21R, CXCR5/CXCL13) is detected in GD thyroid tissues (197), with a positive correlation between the expression of IL-21 mRNA in GD thyroid tissues and the serum levels of autoantibodies. mRNA expression of CXCR5 and CXCL13 is correlated with the number of lymphocytic infiltrates and ectopic GCs in thyroid tissue from patients affected by HT or GD (198).

## Hepatological and Gastroenterological Diseases

### Autoimmune Hepatitis (AIH)

Autoimmune hepatitis displays varied manifestations from asymptomatic, mild chronic hepatitis to acute-onset fulminant liver failure. Studies with murine models of AIH have raised a possible crucial role of Tfh in the disease (199, 200), by showing that dysregulated Tfh cells in the spleen are responsible for the induction of fatal AIH. In humans, percentages of activated cTfh cells (PD-1^+^ or ICOS^+^ Tfh cells) are increased in patients with AIH compared to controls (201, 202). Frequency of activated cTfh cells is positively correlated with serum IgG levels (201, 202) and negatively with serum albumin and serum prothrombin time (201). This population decreases significantly consecutively to prednisolone treatment, with the decrease of serum alanine transferase (201). Serum levels of IL-21 are higher in patients with AIH than in patients with other liver diseases or healthy volunteers (202, 203), correlating with the numbers of cTfh cells. They are associated with the severity of the disease. Finally, they correlate positively with total serum bilirubin levels and negatively with serum albumin (203). Data obtained from immunohistochemistry studies and from flow cytometry on extracted cells from liver biopsies of patients with AIH have been also published: frequencies of activated Tfh cells are significantly increased in the liver from patients and positively correlate with their circulating counterparts in blood of patients (201). A positive correlation of serum IL-21 levels and the grading of necro-inflammatory activity is also described (203).

### Primary Biliary Cholangitis (PBC)

Circulating Tfh (201), ICOS^+^ cTfh (204) and PD1^+^ cTfh cells (205, 206) are increased in patients with PBC as compared to controls, in association with a decrease in Tfr cells and in consequence in the Tfr/Tfh ratio (206). Levels of cTfh cells are even higher than in AIH patients (204) and functional capacities of cTfh cells from PBC patients are greater than those from controls (204). cTfh frequencies correlate with disease severity (204). They are higher in patients with cirrhosis (non-decompensated or decompensated) than in the non-cirrhotic group, and higher in the group of patients with anti-mitochondrial antibodies than in the group without (205). In patients responders to the classic treatment of PBC (ursodeoxycholic acid), there is a decrease in cTfh rates and a trend to a lower production of IL-21 by Tfh cells (204). As in AIH, histological analysis of liver samples reveals an accumulation of PD1^+^Bcl6^+^ Tfh cells around the damaged interlobular bile ducts in PBC with chronic non-suppurative destructive cholangitis; these infiltrating Tfh cells organize follicle-like structures and collocate with B cells around the bile ducts (204). Similar increases of Tfh cells are also present in spleen samples of patients with PBC, as compared to controls (traumatic splenectomy) (204). The aberrant Tfh cell activation in PBC patients can possibly rely on abnormal response toward bacterial antigens stimulation (207).

### Inflammatory Bowel Diseases (IBD)

Circulating Tfh cells have also been studied in patients with IBD [Crohn’s disease (CD) or ulcerative colitis (UC)], showing an increase in their proportions in IBD patients as compared to healthy controls (208, 209), with a significant specific increase in Tfh1 and Tfh17 subsets (209) and a reduction in the frequency of cTfr cells (208). Levels of cTfh cells are associated with symptoms of severity of IBD: they are significantly elevated in the penetrating form of CD, as compared to the inflammatory or stricturing ones (209). In UC, the values of Mayo clinic score (measuring disease severity) and of C-reactive protein positively correlate with cTfh cells and serum IL-21 levels (208). Because Tfh cells may affect the progression of various cancers and because CD patients are at risk of colorectal cancer, the question has been raised to compare cTfh levels in CD patients with or without neoplasia. Patients who developed a colorectal cancer, compared to those who did not, have a significant increase of cTfh cells (209). In colonic tissue sections of patients with UC, proportion of Tfh cells among CD4^+^ T cells is increase (210).

Specific contribution of IL-21 to the pathogenesis of IBD has been studied. It has been observed enhanced serum levels of this cytokine in patients (208), an increase in its expression in UC colonic tissues (210) and interesting findings indicate an important role of CD4^+^ T intestinal lamina propria lymphocytes in the production of IL-21, especially after their activation by IL-12 (211).

## Common Variable Immune Deficiencies (CVID)

Common variable immune deficiencies is the most symptomatic primary immunodeficiency and manifests by recurrent respiratory and gastrointestinal tract infections. This disease is heterogeneous and diagnosis criteria, as defined by European society for immunodeficiencies, include onset after 2 years old, deficit in serum Ig (multiple classes) not explained by other known causes and impaired vaccination responses (212). Impaired B cell differentiation is a hallmark of the disease and, despite normal levels of total B cells in most cases, patients harbor lower levels or absence of smB cells (213). Based on these alterations, the European multicenter trials proposed a classification called EUROClass which hinged on levels of circulating B cells, of smB cells and on the presence of an expansion of transitional B cells or of CD21^low^ cells (214). In more than half of the patients, CVID causes inflammatory disorders, such as lymphoproliferation, granulomatous disease, malignancy, and autoimmunity on the top of the immunodeficiency leading to infection susceptibility (215). These complications cause increased morbidity and mortality. More than 25% of CVID patients have autoimmune complications (215). ITP and autoimmune hemolytic anemia are the most frequent autoimmune disorders but numerous others, such as vitiligo, pernicious anemia, SLE, RA, antiphospholipid syndrome, anti-IgA Ab disease, juvenile idiopathic arthritis, Sjogren’s syndrome, psoriasis, thyroiditis, uveitis, and vasculitis can also be found in CVID patients (215). These observations highlight a paradigm in CVID pathogenesis: despite a defect in B cell differentiation and in serum Ig, patients develop autoantibodies and harbor autoimmune complications. Mechanisms responsible for this paradigm may highlight failures in specific checkpoints for autoreactive B cells and are yet to be clearly identified. Interestingly, Patuzzo et al. (216) have shown an increase in the CD21^low^ population in CVID patients with ITP. CD21^low^ cells may develop under chronic inflammatory conditions from memory B cells and are present in high levels in autoimmune patients including ones affected by ITP, pSS, and SLE. Therefore, one can hypothesize a role of these CD21^low^ smB cells in the development of autoimmune complications observed CVID patients. Nevertheless, further experiments are needed to explore this possibility.

CD4^+^ T cells play a central role in B cell differentiation into memory and Ig-producing cells. It is, therefore, not surprising to observe abnormalities of the T cell compartment in CVID patients. Patients have usually lower levels of CD4^+^ T cells and normal number of CD8^+^ T cells. Genetic defects in the T cell compartment may be detected. One example is the discovery of ICOS mutations in some patients. ICOS has a key role in GC reactions and in smB cell generation and as mentioned earlier, most CVID patients have defects in this B cell population. Grimbacher et al. have identified homologous deletion of *ICOS* genes in CVID patients causing a failure in ICOS expression on T cells (217, 218). These patients have impaired GC formation and defects in class-switching leading to hypogammaglobulinemia and, therefore, defective T cell helping in late B cell differentiation. Combining clinical features of the nine ICOS-deficient patients allow manifestation of the full range of non-infectious related complications. Nevertheless, four of these patients do not have autoimmune diseases. Interestingly, these patients also present lower levels of circulating CXCR5^+^ Tfh cells despite normal numbers of CD4^+^ T cells (15). Among the CD4^+^ T cells, Tfh cells play a critical role in B cell differentiation especially for smB cell differentiation. It seems critical to assess the potential role of these cells in CVID pathogenesis. Our group (submitted manuscript) and others observed an increase of circulating Tfh cells in CVID patients (219–221). Interestingly, patients with non-infectious complications or classified as smB based on the EUROClass harbor increased cTfh (220) which is even more pronounced in the smB- CD21^low^ subgroup (221). Tiller et al. demonstrated in 2007 (222) that the smB cell population (IgG^+^) contains some autoreactive B cells in normal adults. It is then possible that smB cells in CVID patients, despite their low levels, contribute to autoimmunity. Consequently, Tfh cells could be a part of the immune responses leading to autoimmune manifestations observed in CVID patients considering their central role in smB cell differentiation. Moreover, as mentioned earlier, CD21^low^ memory B cells are increased in several autoimmune contexts (216). Still, when we compared patients with autoimmune complications with patients harboring other type of comorbidities, we could not detect any significant differences in levels of Tfh or Tfh subtypes (submitted manuscript). It will be then necessary to perform further experiments to assess the functions of Tfh cells in CVID patients and to evaluate their impact on autoreactive Ab secretion.

Regarding Tfh cell subpopulations, we (submitted manuscript) and others (220, 221) highlight specific increases of the cTfh1 in non-infectious only CVID patient blood. By contrast, cTfh17 cells were decreased. Moreover, Tfh cells from CVID patients expressed elevated levels of IFNγ (220, 221) and sera from patients contained higher concentrations of IFNγ compared to healthy donors (221). Finally, we and Unger et al. showed respectively higher levels of CXCR3 and T-bet, markers of Th1-oriented cells, in SLO from CVID patients. Altogether, these data demonstrated an imbalance in Tfh subpopulations in CVID patients harboring non-infectious complications in favor to cTfh1. These data suggest an IFNγ-enriched environment during B cell differentiation/maturation in CVID patients. Cols et al. have associated elevated IFNγ levels with manifestations of complications in CVID patients and expansion of type 3 innate-lymphoid cells (ILCs) (223). Friedmann et al. (224). confirmed an imbalance in circulating ILC2/ILC3 cells in CVID patients but argued that given the relative abundance of Th1CD4^+^ T cells ILC would not be the main source of IFNγ in CVID. Unger et al. showed that exogenous addition of IFNγ in B/T cocultures reduces IgG and IgA production (221). Still, the impact of IFNγ on CD21^low^ cell generation and/or on autoreactive B cell activation was not directly addressed in CVID and, therefore, is yet to be determined. In conclusion, evidences from the literature strongly suggest a role for Tfh in pathogenesis of the more severe forms of CVID, but not limited to the autoimmune disorders observed in some of these patients. Experiments are still needed to determine Tfh implication in CVID and the possible role of IFNγ in autoimmune manifestations observed in this syndrome.

## Tfh Cells and other Inflammatory and Auto-Immune Diseases

### IgA Nephropathy (IgAN) in Adults

IgA nephropathy is the most common form of primary glomerulonephritis in adults, characterized by mesangial deposition of IgG and IgA in glomeruli. Percentages of CD4^+^CXCR5^+^, CD4^+^CXCR5^+^ICOS^+^, and CD4^+^CXCR5^+^PD1^+^ Tfh, as well as a serum level of IL-21, are increased in patients as compared to controls. Percentages of cTfh cells are negatively correlated with renal functioning (eGFR values) while levels of PD1^+^ Tfh cells are positively correlated with levels of galactose-deficient IgA1 (Gd-IgA1, known to be effector molecules in the pathogenesis of IgAN), as well as 24-h urinary proteins. Treatment with prednisone significantly reduces the frequency of cTfh and levels of serum IL-21 (225).

### Adults Idiopathic Inflammatory Myopathies (IIM)

Idiopathic inflammatory myopathies are a group of heterogeneous chronic autoimmune diseases comprising dermatomyositis and polymyositis, in which muscles are infiltrated by lymphocytes, dendritic cells, and macrophages. Total cTfh cells are expanded in peripheral blood of patients with IIM compared to controls. This expansion is due to the increase of the Tfh2 and Tfh17 subsets. By contrast, levels of Tfh1 cells are decreased (226). The expansion of Tfh2 and Tfh17 is in line with the work published in 2011 by Morita et al., which was the first study describing cTfh cells and their subsets in humans (56). They described a skewing of cTfh cells subsets toward Tfh2 and Tfh17 phenotypes, in relation to disease activity (skin rash, muscular weakness) and frequency of blood plasmablasts in patients with juvenile dermatomyositis (56).

### Systemic Sclerosis (SSc)

Few data are available in literature on the potential role of Tfh cells in SSc pathogenesis. One recent report provides evidence that ICOS^+^ Tfh cells contribute to skin fibrosis (227). T cells with a Tfh phenotype (CD4^+^, CXCR5^+^, and PD-1^+^) and expressing ICOS infiltrate the lesional skin of SSc patients and correlate with dermal fibrosis (assessed by modified Rodnan skin score). Taking advantage of the murine model of sclerodermatous graft-versus-host-disease, authors showed that ICOS^+^ Tfh-like cells contribute to fibrosis *via* IL-21 and matrix metalloproteinase 12. Removal of ICOS^+^ cells ameliorate fibrosis and inhibit dermal inflammation. Finally, by neutralizing specifically IL-21, skin fibrosis is ameliorated as well (227).

## Conclusion

During past years, significant improvements obtained in Tfh cells biology have led to consider their role in various diseases, such as cancers, immunodeficiencies, allergic diseases, but also in autoimmune disorders. First observations published on mouse models, and specially in lupus-prone ones, have demonstrated the pathogenic role of excessive Tfh cell responses in autoimmunity and have paved the way for subsequent research in humans. Since then, the above-mentioned clinical and experimental findings consistently revealed that patients with autoimmune diseases display aberrant Tfh cells responses, with some common features shared across multiple disorders: increased numbers and proportions of total cTfh cells; alteration in the balance of the subsets, with an increase in activated cTfh2 and cTfh17 cell subsets and a decrease in cTfh1 cells; ectopic GC formation in inflamed tissues; association of Tfh cell numbers and activation state with disease activity, levels of autoantibodies and with the response to conventional treatment. However, unlike mouse studies where lymphoid organs can be easily obtained during disease course, many studies performed to date in human auto-immune diseases have largely investigated Tfh cells only in peripheral blood. Like previously mentioned, the exact origin and biology of these cells remain elusive in humans. Whether the information obtained from the analysis of cTfh cells directly reflects the Tfh responses in SLO and/or in inflamed tissues remains to be fully established. Data linking human cTfh cell compartment and Tfh cells in lymphoid organs and/or inflamed tissues are still scarce, functional analysis of cTfh cells (ability to deliver B-cell help) is frequently missing in the published studies and exact nature of Tfh cells present in affected organs requires further studies, like the one published recently by Rao et al. in RA (135).

It is also to be determined if the observed alterations in Tfh cells are causative and/or a consequence of the global immune dysregulation present in these diseases, and for instance regarding B cells. It is established that transmission of signals between Tfh cells and GC B cells is bidirectional. Cognate Tfh—B cell interactions are essential for Tfh cells differentiation and maintenance processes, as B cells serve as APCs and as a source of ICOS-L and cytokines (6, 17, 45, 228–232). This interdependent relationship could possibly be harmful in the context of auto-immunity and this feed-forward loop for Tfh cell help delivered by B cells should be considered with precaution. In fact, if expansion of Tfh cells can provide pathological signals of survival and escape to auto-reactive B cells, the reverse statement could also be true. Autoantigens are easily accessible to B cells during auto-immune reactions and this persistent antigenic availability was shown to favor the activation of Tfh cells in a murine model (45). Besides this, in humans, it was observed that after B cell depleting therapy, in lymph nodes, despite the absence of GC, a population of CD4^+^CXCR5^+^CD57^+^ Tfh cells was still present, suggesting that possibly some resident memory Tfh cells do not require B cells for their maintenance (233, 234).

As a whole, the studies mentioned in this review lend support to the existence of a global increase of the activity of the Tfh lineage in patients with auto-immune and inflammatory disorders and to the more likely association of exaggerated Tfh response with the pathogenesis of human autoimmune diseases. There are still a lot of unanswered and open questions in this field. A better understanding of the biology of these cells, of the events that initiate or sustain their activation is of particular importance, as interference with these processes and their selective inhibition could represent therapeutic options.

## Author Contributions

CR and NG wrote the first draft of the manuscript. NG, MC, DD, CC-B, M-ET, EL, PD and PB wrote sections of the manuscript. PB and CR revised critically the whole work. All authors contributed to manuscript writing, read and approved the submitted version.

## Conflict of Interest Statement

The authors declare that the research was conducted in the absence of any commercial or financial relationships that could be construed as a potential conflict of interest.
